# Postoperative Opioid Receipt After Parotidectomy and Associations With Persistent Opioid Use Disorder

**DOI:** 10.1002/ohn.70025

**Published:** 2025-09-11

**Authors:** Asher C. Park, Milan P. Fehrenbach, Larry W. Wang, Oluwatobiloba Ayo‐Ajibola, Urjeet A. Patel, Sandeep Samant, Katelyn O. Stepan

**Affiliations:** ^1^ Department of Otolaryngology–Head and Neck Surgery Northwestern University Feinberg School of Medicine Chicago Illinois USA; ^2^ College of Medicine University of Kentucky Lexington Kentucky USA; ^3^ Keck School of Medicine University of Southern California Los Angeles California USA

**Keywords:** parotidectomy, persistent opioid use, opioid

## Abstract

**Objective:**

To investigate the association between postoperative opioid receipt and the development of persistent opioid use disorder following parotidectomy.

**Study Design:**

Propensity‐matched retrospective cohort study.

**Setting:**

United States.

**Methods:**

The TriNetX Research Network was queried for adult patients who underwent parotidectomy from 2010 to 2023. Patients with a history of opioid abuse/dependence or underwent surgery with anesthesia within 9 months postoperatively were excluded. The opioid cohort included patients that received hydrocodone, oxycodone, or codeine postoperatively, whereas the nonopioid cohort included patients that did not receive these opioids postoperatively. Cohorts were propensity score matched for patient demographics, psychiatric and preoperative pain diagnoses, as well as for possible procedures performed concurrently alongside parotidectomy. The primary outcome was risk of persistent opioid use, defined as new opioid use 3 to 9 months following surgery. Secondary outcomes included the number of emergency department and office/outpatient visits within 1 and 3 months postoperatively and the risk of opioid abuse/dependence.

**Results:**

After propensity matching, each cohort had 3858 patients. The opioid cohort had an increased risk of developing new persistent opioid use (RR = 1.321, *P* < .001) compared to the nonopioid cohort. Opioid receipt was also associated with increased office/outpatient visits within 1 (*P* < .001) and 3 months (*P* < .001). The number of emergency department visits and risk of opioid abuse/dependence were not significantly different between cohorts.

**Conclusion:**

Opioid prescription following parotidectomy was associated with an increased risk of persistent opioid use disorder and the number of postoperative office visits, emphasizing the need for further investigation of opioid‐sparing analgesia protocols following parotidectomy.

Over the last two decades, opioid overdose‐related deaths have nearly quadrupled, rising from 8.2 to 32.6 deaths per 100,000 people in the United States.^1^ Surgical procedures may be implicated as a major source of opioids with more than 80% of surgical patients being prescribed opioids for postoperative pain management therapy, even after low‐risk surgeries.^2^ More than two‐thirds of patients have been found to have unused opioids postoperatively, suggesting a laxity in current opioid prescribing practices.^3^ Long‐term opioid use may be a consequence of such practices, with 10% of opioid‐naïve patients demonstrating continued opioid use 1 year after low‐pain, short‐stay procedures.^4^ According to Medicare data, otolaryngologists account for nearly 10% of opioid prescriptions.^5^ Despite the frequency of these prescriptions within the specialty, there are few clear guidelines regarding postoperative pain management allowing for large variations in prescribing practices based on surgeon preference and procedure performed.^6^ Furthermore, opioid prescribing patterns have been shown to vary regionally, with otolaryngologists prescribing at higher rates in the Midwest and lower rates in the Northeast.^5^

Approximately 4.6 per 100,000 people undergo parotidectomy annually in the United States.^7^ Postoperative pain following parotidectomy typically peaks within 24 hours, and gradually decreases throughout the week.^8^, ^9^, ^10^ These symptoms have been characterized as mild to moderate and may include neuropathic pain.^11^, ^12^, ^13^ With parotidectomy commonly performed as an outpatient procedure, it remains important to provide adequate analgesia to treat patients' pain following discharge.^14^ However, there is significant variation in the use of opioid analgesics, with different institutions prescribing differing morphine equivalent doses per patient.^15^, ^16^ Currently, there is limited literature describing postoperative opioid‐sparing analgesia regimens following parotidectomy, indicating a lack of consensus regarding best practices.^17^

Previous studies have highlighted institutional differences in opioid prescribing practices following parotidectomy. Postoperative opioid usage was assessed in patients undergoing parotidectomy in a multiphasic study by Dang et al, revealing that patients on average used 5.7 of 15.7 opiate pills prescribed postoperatively.^16^ Another single‐center study evaluating opioid prescribing habits among otolaryngologists found that an average of 22.7 opioid tablets were prescribed to patients following parotidectomy, conflicting with the amount reported in the previous study.^18^ A retrospective cohort study by Rubin et al demonstrated similar findings when comparing mean morphine milligram equivalents (MME) before and after the implementation of a mandatory opioid prescription reporting system (MassPAT).^15^ Through univariate analysis, Rubin et al reported a significant decrease in the amount of opioids prescribed among patients in the MassPAT period, along with lower rates of required refills. These findings underscore the variance in opioid prescribing practices and suggest that parotidectomy patients may be overprescribed opioids. With prior studies describing persistent opioid use (POU) disorder as one of the most common complications following surgery, opioid prescription may contribute to misuse among parotidectomy patients, as well as increased healthcare costs, which have been found to be greater among chronic opioid users.^19^, ^20^, ^21^, ^22^, ^23^, ^24^, ^25^, ^26^, ^27^

Previous literature has defined POU as having 1 or more opioid prescription refills 3 to 9 months postoperatively.^28^, ^29^, ^30^ To date, there are no studies evaluating long‐term opioid use following parotidectomy, making it unclear whether this patient population is at risk of developing POU. The purpose of this study is to assess POU risk among patients undergoing parotidectomy, utilizing a nationally representative large‐claims database. Secondarily, this study evaluates the relationship between postoperative opioid prescription and risk of emergency department (ED) visits, outpatient visits, and diagnosis of opioid dependence or abuse.

## Methods

The TriNetX US Collaborative Network is a national claims database that provides real‐time access to data from electronic medical records of 69 healthcare organizations (HCOs) across the United States. This database is deidentified and is compliant with HIPAA Privacy Rules and therefore was exempt from Institutional Review Board (IRB) review. International Classification of Disease (ICD) codes were used to determine diagnosis, Current Procedural Terminology (CPT) codes were used to define procedures, and RxNorm and Veteran Affairs (VA) Classification codes were used to define medications (Supplemental Appendix 1, available online).

The database was queried on December 14, 2024, for adult patients (≥18 years of age) that underwent parotidectomy from January 1, 2010, to December 11, 2023. Patients with a history of opioid dependence/abuse, opioid receipt within 6 months before parotidectomy, or another surgery with anesthesia within 9 months postoperatively were excluded. The opioid cohort was defined by receipt of oxycodone, hydrocodone, or codeine within 2 days of parotidectomy, similar to prior studies.^31^, ^32^ The nonopioid cohort did not receive these medications within 2 days of parotidectomy. These opioids were selected for this study to include oral opioid medications that patients are commonly prescribed upon discharge, attempting to avoid the inclusion of opioids given to patients immediately in the postoperative recovery unit, such as fentanyl.

The primary outcome of interest was POU, defined as the filling of an opioid prescription 3 to 9 months postoperatively.^28^, ^29^, ^31^ Secondary outcomes included opioid abuse/dependence after 9 months following parotidectomy, as well as the number of ED or office/outpatient visits (not limited to otolaryngology visits) within 30 and 90 days postoperatively. To account for previously established confounding variables, propensity score matching across the cohorts was conducted to match for demographics such as age, race, ethnicity, pain‐related diagnoses, mental health‐related diagnoses, and substance use‐related diagnoses (Supplemental Table S1, available online).^31^, ^33^ Additionally, cohorts were propensity scored matched across procedures that may be performed concurrently with parotidectomy such as neck dissection, free/local flaps, soft/bony tissue resection, facial nerve repair, and temporal bone resection, to mitigate the potential confounding effects of these procedures (Supplemental Table S1, available online). Propensity score matching through TriNetX utilizes logistic regression to calculate propensity scores, or the likelihood of subjects to receive postoperative opioids based on baseline characteristics. Subjects across the two cohorts are then matched using these propensity scores to optimize an equal distribution of baseline covariates that is not significantly statistically different across the cohorts. Cohorts were considered balanced if the mean standardized differences were less than 0.1 for each characteristic.

Cohort demographics before and after matching were described with descriptive and comparative statistics (*t* test and chi‐square as appropriate). The risk ratios (RRs) for patients refilling opioid analgesics 3 to 9 months following parotidectomy and for the diagnosis of opioid abuse/dependence after 9 months post‐op were calculated. The average number of ED and office visits within 30 and 90 days was compared across cohorts using *t* tests. Statistical analyses were performed with TriNetX's built‐in software and R Statistical Software 4.1.2 (R Foundation), with statistical significance set at *α* = .05.

## Results

From 2010 to 2023, a total of 18,357 patients who underwent a parotidectomy were included in this study. Approximately 79% percent of patients received opioids following parotidectomy (n = 14,498). After propensity‐score matching, 3858 parotidectomy patients were included in each cohort. There was no significant difference in mean age among patients in the Opioid (mean ± SD: 58.3 ± 16.8) and nonopioid (58.6 ± 17.0) cohorts (Table 1). The opioid and nonopioid cohorts were distributed similarly across males (49.3%, 49.7%) and females (48.9%, 48.8%). White patients were the majority race in both opioid (65.5%) and nonopioid cohorts (67.6%). Incidence of pain‐related, mental health‐related, and substance use‐related diagnoses among both cohorts is reported in Supplemental Table S1, available online.

**Table 1:** Cohort Demographics With Propensity Score Matching

	Before matching (n = 18,357)				After matching (n = 7716)			
Characteristic	Opioid (n = 14,498)	Nonopioid (n = 3859)	*P*‐value	Std diff.	Opioid (n = 3858)	Nonopioid (n = 3858)	*P*‐value	Std diff.
Age at index, mean ± SD	58.1 ± 16.7	58.6 ± 17.0	.108	0.029	58.3 ± 16.8	58.6 ± 17.0	.422	0.018
Sex								
Female	6717 (46.3%)	1882 (48.8%)	.007	0.049	1888 (48.9%)	1881 (48.8%)	.873	0.004
Male	7374 (50.9%)	1919 (49.7%)	.210	0.023	1902 (49.3%)	1919 (49.7%)	.699	0.009
Unknown gender	407 (2.8%)	58 (1.5%)	<.001	0.090	68 (1.8%)	58 (1.5%)	.369	0.020
Ethnicity								
Not Hispanic or Latino	11,731 (80.9%)	2896 (75.0%)	<.001	0.142	2821 (73.1%)	2896 (75.1%)	.051	0.044
Hispanic or Latino	803 (5.5%)	471 (12.2%)	<.001	0.236	483 (12.5%)	470 (12.2%)	.653	0.010
Race								
White	10,405 (71.8%)	2608 (67.6%)	<.001	0.091	2528 (65.5%)	2608 (67.6%)	.054	0.044
Black or African American	1188 (8.2%)	340 (8.8%)	.218	0.022	346 (9.0%)	340 (8.8%)	.810	0.005
Asian	905 (6.2%)	211 (5.5%)	.074	0.033	227 (5.9%)	211 (5.5%)	.431	0.018
Other race	453 (3.1%)	106 (2.7%)	.225	0.022	130 (3.4%)	106 (2.7%)	.113	0.036
American Indian or Alaska Native	54 (0.4%)	10 (0.3%)	.288	0.020	10 (0.3%)	10 (0.3%)	1.000	<0.001
Native Hawaiian or Pacific Islander	112 (0.8%)	14 (0.4%)	.006	0.055	15 (0.4%)	14 (0.4%)	.852	0.004
Unknown race	1381 (9.5%)	571 (14.8%)	<.001	0.162	603 (15.6%)	570 (14.8%)	.295	0.024

Abbreviation: Std diff., mean standard difference.

Risk of POU 3 to 9 months postoperatively increased by 32% among patients who received an opioid prescription compared to those who did not (RR: 1.321 [1.150, 1.517], *P* < .001) (Table 2). Within 30 days following parotidectomy, there was a significantly increased average number of office/outpatient visits among patients who received an opioid prescription (0.511 ± 0.93) compared to that of the nonopioid cohort (0.323 ± 0.75) (*P* < .001) (Table 3). Similarly, opioid prescription was associated with an increased average number of office/outpatient visits within 90 days (0.957 ± 1.79) postoperatively compared to the nonopioid cohort (0.68 ± 1.44) (*P* < .001). There were no significant differences in the average number of ED visits 30 and 90 days postoperatively between both cohorts. Similarly, there was no significant difference in risk of opioid abuse or dependence among opioid and nonopioid cohorts after 9 months postoperatively (Table 2).

**Table 2:** Comparison of Persistent Opioid Use (3‐9 Months Postoperatively) and Opioid Abuse or Dependence (After 9 Months Postoperatively) Between Patients Who Received Oxycodone, Hydrocodone, or Codeine Within 0 to 2 Days After Parotidectomy Versus Those Who Did Not

	After matching patients (N = 7716)			
	Opioid	Nonopioid		
Outcome	(n = 3858)	(n = 3858)	RR (95% CI)	*P*‐value
Persistent opioid use (3‐9 mo post‐op)	420 (11.10%)	318 (8.40%)	1.321 (1.150, 1.517)	<.001
Opioid abuse or dependence after 6 mo post‐op	14 (0.37%)	13 (0.34%)	1.077 (0.507, 2.288)	.847

Abbreviations: CI, confidence interval; RR, risk ratio.

**Table 3:** Comparison of the Average Number of Emergency Department (ED) and Office Visits Within 30 to 90 Days Postoperatively Between Patients Who Received Oxycodone, Hydrocodone, or Codeine Within 0 to 2 Days After Parotidectomy Versus Those Who Did Not

	After matching patients, no. (%) (n = 7716)				
	Opioid (n = 3858)		Nonopioid (n = 3858)		
Outcome	Mean number of visits	Std dev.	Mean number of visits	Std dev.	*P*‐value
ED visits within 30 d post‐op	0.029	0.193	0.036	0.217	.061
ED visits within 90 d post‐op	0.049	0.265	0.053	0.314	.173
Office/outpatient visits within 30 d post‐op	0.511	0.930	0.323	0.749	<.001
Office/outpatient visits within 90 d post‐op	0.957	1.793	0.678	1.439	<.001

Abbreviation: Std dev., standard deviation.

## Discussion

The rise in opioid‐associated deaths in recent years raises significant concerns regarding opioid stewardship, especially in the context of postoperative pain management. Despite the widespread use of opioids following parotidectomy, the risks of POU development following postoperative opioid prescription remain poorly understood. This study sought to fill this gap in the literature through a propensity‐matched retrospective analysis using the TriNetX US Collaborative Network. This study found that opioid prescription following parotidectomy was associated with a significantly increased risk of POU. Although ED visits within 30 and 90 days postoperation were not significantly associated with opioid prescription, opioid receipt was associated with an increased average number of office/outpatient visits within 30 and 90 days postoperation, likely increasing healthcare resource utilization among opioid recipients following parotidectomy. Lastly, this study did not find a significant increase in opioid abuse or dependence after 9 months postoperation. However, there remains a significant need for optimization and consensus regarding nonopioid pain management considering the association between postoperative opioid prescription dispersion and the present opioid crisis.^34^

The increased likelihood of POU observed in this study aligns with existing literature. Previous studies examining opioid‐naïve surgical patients have estimated the risk of postoperative POU to be 3% to 15% for major and minor surgeries.^35^, ^36^, ^37^ For parotid surgery, Biskup et al reported postoperative opioid prescription in 88% of patients.^38^ Although there is a dearth of literature regarding rates of opioid prescription refills following parotidectomy, overprescription of opioids may be a contributing factor to the increased risk of POU seen in this study. In a single‐institution study of elective otolaryngologic procedures, Jawad et al reported the highest opioid utilization among parotidectomy and tonsillectomy procedures.^39^ However, studies from Koch et al and Dang et al have reported that the majority of parotidectomy patients experience minimal pain, with postoperative pain peaking one day postoperation,^16^, ^40^ which likely contributes to the 65% of total opioids remaining unused.^16^ Lewis et al surveyed patients with known opioid risk factors and found that 34% of patients prescribed opioids reported engaging in diversion or sharing of unused opioids with friends and family at least once.^41^ The reported substantial quantities of unused opioids following parotidectomy emphasize the need for otolaryngologists to consider minimizing opioid prescriptions while optimizing opioid‐sparing pain management to prevent the unnecessary spread and inappropriate usage of postoperative opioids.

Due to increased emphasis on opioid stewardship, recent American Academy of Otolaryngology–Head and Neck Surgery Foundation guidelines have highlighted the importance of minimizing or avoiding opioid prescriptions following common otolaryngology procedures.^42^ Specifically, the action statement advocating for nonopioid analgesia as first‐line management of postoperative pain was designated as a “strong recommendation.” Currently, there are no established guidelines for standardized nonopioid pain management regimens in parotidectomy. However, Oltman et al proposed a pain management regimen for this procedure that included preoperative analgesia with acetaminophen (1000 mg), gabapentin (100‐300 mg, based on age and weight), and meloxicam (7.5 mg).^17^ Postoperative analgesia comprised of alternating oral doses of ibuprofen (600 mg) and acetaminophen (500 mg) every 6 hours. This regimen demonstrated low resting and peak pain scores following surgery, and 61% of patients avoided opioid use. Although the use of nonsteroidal anti‐inflammatory drugs (NSAIDs) in perioperative pain management may raise concern due to perceived risk of bleeding, Oltman et al did not report any events related to bleeding or hematoma.^17^ The regimen proposed by this group also corroborates existing literature suggesting that nonopioid pain management regimens may adequately mitigate post‐parotidectomy pain.^43^ As such, there is a growing body of evidence that questions the notion that opioids should be utilized primarily for pain relief following parotidectomy. This emphasizes the need for further studies establishing effective opioid‐sparing analgesia regimen guidelines following parotidectomy.

This study also found that postoperative opioid prescription was significantly associated with increased outpatient/office visits at 30 and 90 days post‐surgery. Although opioid prescriptions cannot be directly implicated as the cause of increased unplanned office visits, these results do at least suggest that avoiding opioids does not correlate with significantly increased unplanned outpatient/office visits. This trend is consistent with existing literature within the general surgery setting, where reduced opioid and opioid‐sparing modalities for pain management have been associated with sufficient pain management without increasing the number of unplanned office visits.^44^, ^45^ Additionally, prior studies have found that patients with a history of opioid abuse, overdose, or dependence have an increase of $14,810 in direct medical costs per patient.^46^ Our study's finding of increased risk of post‐op office visits among opioid recipients seems to align with this finding of increased healthcare utilization and burden among patients with notable opioid use histories. Reducing opioid prescriptions for parotidectomy patients may have implications in reducing healthcare costs and burden.

Limitations of this study are those consistent with retrospective database studies. This study relies on the accuracy of diagnosis and procedure codes, which may vary by physician documentation practices. The reliance on billing codes also limits the detail available for variables of interest. For instance, TriNetX is not able to capture the opioid dosage or frequency, preoperative analgesic plan details, nor the reasons for ED and office/outpatient visits—making it difficult to delineate between planned and unplanned visits. These limitations prevent us from drawing definitive conclusions about the relationship between opioid prescriptions and healthcare visits as well as the potential role of preoperative analgesic plans in regards to pain management for parotidectomy. Additionally, insurance status and unrelated events like injury or painful events were two variables that were not captured by the database, which may influence the incidence of POU. Future studies should subsequently explore with more granularity, the role of preoperative pain management as well as the risk of healthcare utilization in the context of parotidectomy. Additionally, further research should be conducted to evaluate the dose‐dependent risk of postoperative opioids on the development of POU.

## Conclusion

This study's results demonstrate that opioid prescriptions following parotidectomy are significantly associated with increased risk of POU and office/outpatient visits 30 and 90 days post‐surgery, emphasizing the need for caution when selecting a pain management modality following parotidectomy. Future studies should further establish effective opioid‐sparing analgesic regimens for postoperative parotidectomy pain.

## Author Contributions

**Asher C. Park,** conceptualization, data curation, formal analysis, investigation, methodology, project administration, supervision, visualization, writing—original draft preparation, writing—review and editing; **Milan P. Fehrenbach,** data curation, formal analysis, investigation, visualization, writing—original draft preparation, writing—review and editing; **Larry W. Wang,** data curation, formal analysis, investigation, visualization, writing—original draft preparation, writing—review and editing; **Oluwatobiloba Ayo‐Ajibola,** data curation, formal analysis, investigation, visualization, writing—original draft preparation, writing—review and editing; U**rjeet A. Patel,** conceptualization, investigation, methodology, project administration, supervision, writing—review and editing; **Sandeep Samant,** conceptualization, investigation, methodology, project administration, supervision, writing—review and editing; **Katelyn O. Stepan,** conceptualization, investigation, methodology, project administration, supervision, writing—review and editing.

## Disclosures

### Competing interests

The authors declare no conflicts of interest.

### Funding source

The authors received no financial support for the research, authorship, and/or publication of this article.

## Supporting information

Supporting Information.

Supporting Information.
