# Supplementary material for: Postoperative Opioid Receipt After Parotidectomy and Associations With Persistent Opioid Use Disorder
Source: Otolaryngol Head Neck Surg. 2025 Sep 11;173(5):1131–7. doi: 10.1002/ohn.70025 (PMC12574618; doi:10.1002/ohn.70025)
Supplement: Supplementary file 1 — Supporting Information. [file OHN-173-1131-s002.docx]

**Supplemental Materials**

Supplemental Table 1: Cohort diagnoses and procedures pre- and post-propensity matching

| **Cohort 1 (N = 14,498) and cohort 2 (N = 3,859) characteristics before propensity score matching** | | | | | | | | | |
| --- | --- | --- | --- | --- | --- | --- | --- | --- | --- |
|  | **Demographics** | | | | | | | | |
|  |  | Cohort | |  | Mean ± SD | Patients | % of Cohort | P-Value | Std diff. |
|  |  | 1 2 | AI | Age at Index | 58.1 +/- 16.7 58.6 +/- 17.0 | 14,498 3,859 | 100% 100% | 0.108 | 0.029 |
|  |  | 1 2 | F | Female |  | 6,717 1,882 | 46.3% 48.8% | 0.007 | 0.049 |
|  |  | 1 2 | 2054-5 | Black or African American |  | 1,188 340 | 8.2% 8.8% | 0.218 | 0.022 |
|  |  | 1 2 | M | Male |  | 7,374 1,919 | 50.9% 49.7% | 0.210 | 0.023 |
|  |  | 1 2 | 2106-3 | White |  | 10,405 2,608 | 71.8% 67.6% | <0.001 | 0.091 |
|  |  | 1 2 | 1002-5 | American Indian or Alaska Native |  | 54 10 | 0.4% 0.3% | 0.288 | 0.020 |
|  |  | 1 2 | UNK | Unknown Race |  | 1,381 571 | 9.5% 14.8% | <0.001 | 0.162 |
|  |  | 1 2 | 2076-8 | Native Hawaiian or Other Pacific Islander |  | 112 14 | 0.8% 0.4% | 0.006 | 0.055 |
|  |  | 1 2 | UN | Unknown Gender |  | 407 58 | 2.8% 1.5% | <0.001 | 0.090 |
|  |  | 1 2 | 2186-5 | Not Hispanic or Latino |  | 11,731 2,896 | 80.9% 75.0% | <0.001 | 0.142 |
|  |  | 1 2 | 2135-2 | Hispanic or Latino |  | 803 471 | 5.5% 12.2% | <0.001 | 0.236 |
|  |  | 1 2 | 2131-1 | Other Race |  | 453 106 | 3.1% 2.7% | 0.225 | 0.022 |
|  |  | 1 2 | 2028-9 | Asian |  | 905 211 | 6.2% 5.5% | 0.074 | 0.033 |
|  | **Diagnosis** | | | | | | | | |
|  |  | Cohort | |  | Mean ± SD | Patients | % of Cohort | P-Value | Std diff. |
|  |  | 1 2 | M15-M19 | Osteoarthritis |  | 1,869 463 | 12.9% 12.0% | 0.139 | 0.027 |
|  |  | 1 2 | M54 | Dorsalgia |  | 2,376 616 | 16.4% 16.0% | 0.525 | 0.012 |
|  |  | 1 2 | M25.5 | Pain in joint |  | 2,465 593 | 17.0% 15.4% | 0.015 | 0.044 |
|  |  | 1 2 | G89 | Pain, not elsewhere classified |  | 1,174 281 | 8.1% 7.3% | 0.095 | 0.031 |
|  |  | 1 2 | M79.6 | Pain in limb, hand, foot, fingers and toes |  | 1,635 402 | 11.3% 10.4% | 0.131 | 0.028 |
|  |  | 1 2 | R07.8 | Other chest pain |  | 865 221 | 6.0% 5.7% | 0.575 | 0.010 |
|  |  | 1 2 | R10.2 | Pelvic and perineal pain |  | 494 157 | 3.4% 4.1% | 0.048 | 0.035 |
|  |  | 1 2 | R51 | Headache |  | 1,084 272 | 7.5% 7.0% | 0.366 | 0.017 |
|  |  | 1 2 | G47.0 | Insomnia |  | 531 107 | 3.7% 2.8% | 0.007 | 0.050 |
|  |  | 1 2 | M79.1 | Myalgia |  | 472 125 | 3.3% 3.2% | 0.959 | 0.001 |
|  |  | 1 2 | G44 | Other headache syndromes |  | 556 183 | 3.8% 4.7% | 0.011 | 0.045 |
|  |  | 1 2 | M79.7 | Fibromyalgia |  | 316 89 | 2.2% 2.3% | 0.634 | 0.009 |
|  |  | 1 2 | H93.1 | Tinnitus |  | 343 89 | 2.4% 2.3% | 0.828 | 0.004 |
|  |  | 1 2 | G43 | Migraine |  | 487 116 | 3.4% 3.0% | 0.274 | 0.020 |
|  |  | 1 2 | K30 | Functional dyspepsia |  | 127 21 | 0.9% 0.5% | 0.041 | 0.040 |
|  |  | 1 2 | M26.6 | Temporomandibular joint disorders |  | 199 47 | 1.4% 1.2% | 0.458 | 0.014 |
|  |  | 1 2 | F30-F39 | Mood [affective] disorders |  | 1,435 308 | 9.9% 8.0% | <0.001 | 0.067 |
|  |  | 1 2 | F41 | Other anxiety disorders |  | 1,381 297 | 9.5% 7.7% | <0.001 | 0.065 |
|  |  | 1 2 | F43 | Reaction to severe stress, and adjustment disorders |  | 393 102 | 2.7% 2.6% | 0.818 | 0.004 |
|  |  | 1 2 | F90 | Attention-deficit hyperactivity disorders |  | 108 25 | 0.7% 0.6% | 0.527 | 0.012 |
|  |  | 1 2 | F60-F69 | Disorders of adult personality and behavior |  | 37 14 | 0.3% 0.4% | 0.259 | 0.019 |
|  |  | 1 2 | F20-F29 | Schizophrenia, schizotypal, delusional, and other non-mood psychotic disorders |  | 89 22 | 0.6% 0.6% | 0.755 | 0.006 |
|  |  | 1 2 | F10-F19 | Mental and behavioral disorders due to psychoactive substance use |  | 2,044 503 | 14.1% 13.0% | 0.089 | 0.031 |
|  |  | 1 2 | F17 | Nicotine dependence |  | 1,822 439 | 12.6% 11.4% | 0.045 | 0.037 |
|  |  | 1 2 | F10 | Alcohol related disorders |  | 283 72 | 2.0% 1.9% | 0.730 | 0.006 |
|  |  | 1 2 | F12 | Cannabis related disorders |  | 113 20 | 0.8% 0.5% | 0.089 | 0.033 |
|  |  | 1 2 | F19 | Other psychoactive substance related disorders |  | 60 19 | 0.4% 0.5% | 0.508 | 0.012 |
|  |  | 1 2 | F14 | Cocaine related disorders |  | 39 10 | 0.3% 0.3% | 0.916 | 0.002 |
|  |  | 1 2 | F15 | Other stimulant related disorders |  | 23 10 | 0.2% 0.3% | 0.190 | 0.022 |
|  |  | 1 2 | F18 | Inhalant related disorders |  | 25 10 | 0.2% 0.3% | 0.273 | 0.019 |
|  | **Procedure** | | | | | | | | |
|  |  | Cohort | |  | Mean ± SD | Patients | % of Cohort | P-Value | Std diff. |
|  |  | 1 2 | 38700 | Suprahyoid lymphadenectomy |  | 10 0 | 0.1% 0% | 0.103 | 0.037 |
|  |  | 1 2 | 38720 | Cervical lymphadenectomy (complete) |  | 10 0 | 0.1% 0% | 0.103 | 0.037 |
|  |  | 1 2 | 38724 | Cervical lymphadenectomy (modified radical neck dissection) |  | 49 10 | 0.3% 0.3% | 0.442 | 0.014 |
|  |  | 1 2 | 20969 | Free osteocutaneous flap with microvascular anastomosis; other than iliac crest, metatarsal, or great toe |  | 10 10 | 0.1% 0.3% | 0.001 | 0.047 |
|  |  | 1 2 | 20955 | Bone graft with microvascular anastomosis; fibula |  | 0 10 | 0% 0.3% | <0.001 | 0.072 |
|  |  | 1 2 | 15758 | Free fascial flap with microvascular anastomosis |  | 10 0 | 0.1% 0% | 0.103 | 0.037 |
|  |  | 1 2 | 15756 | Free muscle or myocutaneous flap with microvascular anastomosis |  | 10 10 | 0.1% 0.3% | 0.001 | 0.047 |
|  |  | 1 2 | 15757 | Free skin flap with microvascular anastomosis |  | 10 10 | 0.1% 0.3% | 0.001 | 0.047 |
|  |  | 1 2 | 1003520 | Graft for facial nerve paralysis |  | 10 0 | 0.1% 0% | 0.103 | 0.037 |
|  |  | 1 2 | 15740 | Flap; island pedicle requiring identification and dissection of an anatomically named axial vessel |  | 10 10 | 0.1% 0.3% | 0.001 | 0.047 |
|  |  | 1 2 | 00QM0ZZ | Repair Facial Nerve, Open Approach |  | 10 0 | 0.1% 0% | 0.103 | 0.037 |
|  |  | 1 2 | 1009692 | Suture of facial nerve |  | 10 0 | 0.1% 0% | 0.103 | 0.037 |
|  |  | 1 2 | 64742 | Transection or avulsion of; facial nerve, differential or complete |  | 10 10 | 0.1% 0.3% | 0.001 | 0.047 |
|  |  | 1 2 | 21025 | Excision of bone (eg, for osteomyelitis or bone abscess); mandible |  | 10 0 | 0.1% 0% | 0.103 | 0.037 |
|  |  | 1 2 | 21026 | Excision of bone (eg, for osteomyelitis or bone abscess); facial bone(s) |  | 10 0 | 0.1% 0% | 0.103 | 0.037 |
|  |  | 1 2 | 21029 | Removal by contouring of benign tumor of facial bone (eg, fibrous dysplasia) |  | 10 0 | 0.1% 0% | 0.103 | 0.037 |
|  |  | 1 2 | 21030 | Excision of benign tumor or cyst of maxilla or zygoma by enucleation and curettage |  | 10 0 | 0.1% 0% | 0.103 | 0.037 |
|  |  | 1 2 | 21040 | Excision of benign tumor or cyst of mandible, by enucleation and/or curettage |  | 10 0 | 0.1% 0% | 0.103 | 0.037 |
|  |  | 1 2 | 21047 | Excision of benign tumor or cyst of mandible; requiring extra-oral osteotomy and partial mandibulectomy (eg, locally aggressive or destructive lesion[s]) |  | 10 0 | 0.1% 0% | 0.103 | 0.037 |
|  |  | 1 2 | 21034 | Excision of malignant tumor of maxilla or zygoma |  | 10 0 | 0.1% 0% | 0.103 | 0.037 |
|  |  | 1 2 | 21031 | Excision of torus mandibularis |  | 10 0 | 0.1% 0% | 0.103 | 0.037 |
|  |  | 1 2 | 61595 | Transtemporal approach to posterior cranial fossa, jugular foramen or midline skull base, including mastoidectomy, decompression of sigmoid sinus and/or facial nerve, with or without mobilization |  | 10 10 | 0.1% 0.3% | 0.001 | 0.047 |
|  |  | 1 2 | 1009212 | Resection or excision of neoplastic, vascular or infectious lesion of infratemporal fossa, parapharyngeal space, petrous apex |  | 11 10 | 0.1% 0.3% | 0.003 | 0.045 |
|  |  | 1 2 | 1009208 | Resection or excision of neoplastic, vascular or infectious lesion of base of anterior cranial fossa |  | 10 0 | 0.1% 0% | 0.103 | 0.037 |
|  |  | 1 2 | 61590 | Infratemporal pre-auricular approach to middle cranial fossa (parapharyngeal space, infratemporal and midline skull base, nasopharynx), with or without disarticulation of the mandible, including parotidectomy, craniotomy, decompression and/or mobilization of the facial nerve and/or petrous carotid artery |  | 10 10 | 0.1% 0.3% | 0.001 | 0.047 |
|  |  | 1 2 | 61591 | Infratemporal post-auricular approach to middle cranial fossa (internal auditory meatus, petrous apex, tentorium, cavernous sinus, parasellar area, infratemporal fossa) including mastoidectomy, resection of sigmoid sinus, with or without decompression and/or mobilization of contents of auditory canal or petrous carotid artery |  | 10 0 | 0.1% 0% | 0.103 | 0.037 |
|  |  | 1 2 | 1003258 | Excision, malignant lesion including margins, face, ears, eyelids, nose, lips |  | 129 43 | 0.9% 1.1% | 0.198 | 0.023 |
| **Cohort 1 (N = 3,858) and cohort 2 (N = 3,858) characteristics after propensity score matching** | | | | | | | | | |
|  | **Diagnosis** | | | | | | | | |
|  |  | Cohort | |  | Mean ± SD | Patients | % of Cohort | P-Value | Std diff. |
|  |  | 1 2 | M15-M19 | Osteoarthritis |  | 388 463 | 10.1% 12.0% | 0.006 | 0.062 |
|  |  | 1 2 | M54 | Dorsalgia |  | 519 616 | 13.5% 16.0% | 0.002 | 0.071 |
|  |  | 1 2 | M25.5 | Pain in joint |  | 501 593 | 13.0% 15.4% | 0.003 | 0.068 |
|  |  | 1 2 | G89 | Pain, not elsewhere classified |  | 251 281 | 6.5% 7.3% | 0.178 | 0.031 |
|  |  | 1 2 | M79.6 | Pain in limb, hand, foot, fingers and toes |  | 350 402 | 9.1% 10.4% | 0.046 | 0.045 |
|  |  | 1 2 | R07.8 | Other chest pain |  | 192 221 | 5.0% 5.7% | 0.142 | 0.033 |
|  |  | 1 2 | R10.2 | Pelvic and perineal pain |  | 132 157 | 3.4% 4.1% | 0.134 | 0.034 |
|  |  | 1 2 | R51 | Headache |  | 233 272 | 6.0% 7.1% | 0.073 | 0.041 |
|  |  | 1 2 | G47.0 | Insomnia |  | 94 107 | 2.4% 2.8% | 0.353 | 0.021 |
|  |  | 1 2 | M79.1 | Myalgia |  | 96 125 | 2.5% 3.2% | 0.048 | 0.045 |
|  |  | 1 2 | G44 | Other headache syndromes |  | 156 183 | 4.0% 4.7% | 0.134 | 0.034 |
|  |  | 1 2 | M79.7 | Fibromyalgia |  | 68 89 | 1.8% 2.3% | 0.090 | 0.039 |
|  |  | 1 2 | H93.1 | Tinnitus |  | 79 89 | 2.0% 2.3% | 0.435 | 0.018 |
|  |  | 1 2 | G43 | Migraine |  | 89 116 | 2.3% 3.0% | 0.056 | 0.044 |
|  |  | 1 2 | K30 | Functional dyspepsia |  | 19 21 | 0.5% 0.5% | 0.751 | 0.007 |
|  |  | 1 2 | M26.6 | Temporomandibular joint disorders |  | 51 47 | 1.3% 1.2% | 0.684 | 0.009 |
|  |  | 1 2 | F30-F39 | Mood [affective] disorders |  | 275 308 | 7.1% 8.0% | 0.155 | 0.032 |
|  |  | 1 2 | F41 | Other anxiety disorders |  | 252 297 | 6.5% 7.7% | 0.046 | 0.045 |
|  |  | 1 2 | F43 | Reaction to severe stress, and adjustment disorders |  | 87 102 | 2.3% 2.6% | 0.269 | 0.025 |
|  |  | 1 2 | F90 | Attention-deficit hyperactivity disorders |  | 23 25 | 0.6% 0.6% | 0.772 | 0.007 |
|  |  | 1 2 | F60-F69 | Disorders of adult personality and behavior |  | 11 14 | 0.3% 0.4% | 0.548 | 0.014 |
|  |  | 1 2 | F20-F29 | Schizophrenia, schizotypal, delusional, and other non-mood psychotic disorders |  | 20 22 | 0.5% 0.6% | 0.757 | 0.007 |
|  |  | 1 2 | F10-F19 | Mental and behavioral disorders due to psychoactive substance use |  | 418 503 | 10.8% 13.0% | 0.003 | 0.068 |
|  |  | 1 2 | F17 | Nicotine dependence |  | 361 439 | 9.4% 11.4% | 0.004 | 0.066 |
|  |  | 1 2 | F10 | Alcohol related disorders |  | 63 72 | 1.6% 1.9% | 0.435 | 0.018 |
|  |  | 1 2 | F12 | Cannabis related disorders |  | 20 20 | 0.5% 0.5% | 1 | <0.001 |
|  |  | 1 2 | F19 | Other psychoactive substance related disorders |  | 15 19 | 0.4% 0.5% | 0.492 | 0.016 |
|  |  | 1 2 | F14 | Cocaine related disorders |  | 10 10 | 0.3% 0.3% | 1 | <0.001 |
|  |  | 1 2 | F15 | Other stimulant related disorders |  | 10 10 | 0.3% 0.3% | 1 | <0.001 |
|  |  | 1 2 | F18 | Inhalant related disorders |  | 10 10 | 0.3% 0.3% | 1 | <0.001 |
|  | **Procedure** | | | | | | | | |
|  |  | Cohort | |  | Mean ± SD | Patients | % of Cohort | P-Value | Std diff. |
|  |  | 1 2 | 38700 | Suprahyoid lymphadenectomy |  | 0 0 | 0% 0% | -- | -- |
|  |  | 1 2 | 38720 | Cervical lymphadenectomy (complete) |  | 10 0 | 0.3% 0% | 0.002 | 0.072 |
|  |  | 1 2 | 38724 | Cervical lymphadenectomy (modified radical neck dissection) |  | 11 10 | 0.3% 0.3% | 0.827 | 0.005 |
|  |  | 1 2 | 20969 | Free osteocutaneous flap with microvascular anastomosis; other than iliac crest, metatarsal, or great toe |  | 0 10 | 0% 0.3% | 0.002 | 0.072 |
|  |  | 1 2 | 20955 | Bone graft with microvascular anastomosis; fibula |  | 0 10 | 0% 0.3% | 0.002 | 0.072 |
|  |  | 1 2 | 15758 | Free fascial flap with microvascular anastomosis |  | 0 0 | 0% 0% | -- | -- |
|  |  | 1 2 | 15756 | Free muscle or myocutaneous flap with microvascular anastomosis |  | 10 10 | 0.3% 0.3% | 1 | <0.001 |
|  |  | 1 2 | 15757 | Free skin flap with microvascular anastomosis |  | 10 10 | 0.3% 0.3% | 1 | <0.001 |
|  |  | 1 2 | 1003520 | Graft for facial nerve paralysis |  | 0 0 | 0% 0% | -- | -- |
|  |  | 1 2 | 15740 | Flap; island pedicle requiring identification and dissection of an anatomically named axial vessel |  | 10 10 | 0.3% 0.3% | 1 | <0.001 |
|  |  | 1 2 | 00QM0ZZ | Repair Facial Nerve, Open Approach |  | 0 0 | 0% 0% | -- | -- |
|  |  | 1 2 | 1009692 | Suture of facial nerve |  | 0 0 | 0% 0% | -- | -- |
|  |  | 1 2 | 64742 | Transection or avulsion of; facial nerve, differential or complete |  | 0 10 | 0% 0.3% | 0.002 | 0.072 |
|  |  | 1 2 | 21025 | Excision of bone (eg, for osteomyelitis or bone abscess); mandible |  | 0 0 | 0% 0% | -- | -- |
|  |  | 1 2 | 21026 | Excision of bone (eg, for osteomyelitis or bone abscess); facial bone(s) |  | 0 0 | 0% 0% | -- | -- |
|  |  | 1 2 | 21029 | Removal by contouring of benign tumor of facial bone (eg, fibrous dysplasia) |  | 0 0 | 0% 0% | -- | -- |
|  |  | 1 2 | 21030 | Excision of benign tumor or cyst of maxilla or zygoma by enucleation and curettage |  | 0 0 | 0% 0% | -- | -- |
|  |  | 1 2 | 21040 | Excision of benign tumor or cyst of mandible, by enucleation and/or curettage |  | 10 0 | 0.3% 0% | 0.002 | 0.072 |
|  |  | 1 2 | 21047 | Excision of benign tumor or cyst of mandible; requiring extra-oral osteotomy and partial mandibulectomy (eg, locally aggressive or destructive lesion[s]) |  | 0 0 | 0% 0% | -- | -- |
|  |  | 1 2 | 21034 | Excision of malignant tumor of maxilla or zygoma |  | 0 0 | 0% 0% | -- | -- |
|  |  | 1 2 | 21031 | Excision of torus mandibularis |  | 0 0 | 0% 0% | -- | -- |
|  |  | 1 2 | 61595 | Transtemporal approach to posterior cranial fossa, jugular foramen or midline skull base, including mastoidectomy, decompression of sigmoid sinus and/or facial nerve, with or without mobilization |  | 10 10 | 0.3% 0.3% | 1 | <0.001 |
|  |  | 1 2 | 1009212 | Resection or excision of neoplastic, vascular or infectious lesion of infratemporal fossa, parapharyngeal space, petrous apex |  | 10 10 | 0.3% 0.3% | 1 | <0.001 |
|  |  | 1 2 | 1009208 | Resection or excision of neoplastic, vascular or infectious lesion of base of anterior cranial fossa |  | 10 0 | 0.3% 0% | 0.002 | 0.072 |
|  |  | 1 2 | 61590 | Infratemporal pre-auricular approach to middle cranial fossa (parapharyngeal space, infratemporal and midline skull base, nasopharynx), with or without disarticulation of the mandible, including parotidectomy, craniotomy, decompression and/or mobilization of the facial nerve and/or petrous carotid artery |  | 10 10 | 0.3% 0.3% | 1 | <0.001 |
|  |  | 1 2 | 61591 | Infratemporal post-auricular approach to middle cranial fossa (internal auditory meatus, petrous apex, tentorium, cavernous sinus, parasellar area, infratemporal fossa) including mastoidectomy, resection of sigmoid sinus, with or without decompression and/or mobilization of contents of auditory canal or petrous carotid artery |  | 10 0 | 0.3% 0% | 0.002 | 0.072 |
|  |  | 1 2 | 1003258 | Excision, malignant lesion including margins, face, ears, eyelids, nose, lips |  | 41 43 | 1.1% 1.1% | 0.826 | 0.005 |
