# Supplementary material for: Postoperative Opioid Receipt After Parotidectomy and Associations With Persistent Opioid Use Disorder
Source: Otolaryngol Head Neck Surg. 2025 Sep 11;173(5):1131–7. doi: 10.1002/ohn.70025 (PMC12574618; doi:10.1002/ohn.70025)
Supplement: Supplementary file 2 — Supporting Information. [file OHN-173-1131-s001.docx]

**APPENDIX**

| **Diagnosis/Procedure** | **Code (ICD-10, CPT, RxNorm, VA Classification)** |
| --- | --- |
| **Parotidectomy Procedures** | |
| Excision of parotid tumor or parotid gland; lateral lobe, without nerve dissection | 42410 |
| Excision of parotid tumor or parotid gland; lateral lobe, with dissection and preservation of facial nerve | 42415 |
| Excision of parotid tumor or parotid gland; total, en bloc removal with sacrifice of facial nerve | 42425 |
| Excision of parotid tumor or parotid gland; total, with dissection and preservation of facial nerve | 42420 |
| **Opioid Abuse/Dependence** | |
| Opioid Abuse | F11.1 |
| Opioid Dependence | F11.2 |
| **Surgery with Anesthesia** | |
| Surgery | 1003143 |
| Anesthesia | 1002796 |
| **Opioids** | |
| Hydrocodone | 5489 |
| Codeine | 2670 |
| Oxycodone | 7804 |
| **Opioid Analgesics** | |
| Opioid Analgesics | CN101 |
| **Emergency Department and Office/Outpatient Visits** | |
| Emergency Department Services | 1013711 |
| Office or Other Outpatient Services | 1013626 |
